# Antioxidant Solution in Combination with Angiotensin-(1-7) Provides Myocardial Protection in Langendorff-Perfused Rat Hearts

**DOI:** 10.1155/2020/2862631

**Published:** 2020-07-30

**Authors:** Michaela Andrä, Miriam Russ, Susanne Jauk, Mariana Lamacie, Ingrid Lang, Robert Arnold, Iva Brcic, Robson Santos, Reinhold Wintersteiger, Astrid Ortner

**Affiliations:** ^1^Department of Cardio-Thoracic and Vascular Surgery, Klinikum Klagenfurt am Wörthersee, 9020 Klagenfurt, Austria; ^2^Institute of Pharmaceutical Sciences, Department of Pharmaceutical Chemistry, University of Graz, 8010 Graz, Austria; ^3^Department of Medicine, Division of Cardiology, University of Ottawa Heart Institute, Ottawa, Ontario, Canada K1Y4W7; ^4^Division of Cell Biology, Histology and Embryology, Gottfried Schatz Research Center, Medical University of Graz, 8010 Graz, Austria; ^5^Gottfried Schatz Research Center-Biophysics, Medical University of Graz, 8010 Graz, Austria; ^6^Diagnostic & Research Institute of Pathology, Medical University of Graz, 8010 Graz, Austria; ^7^Institute of Physiology, Laboratory of Hypertension, University of Minas Gerais, Belo Horizonte, MG 31270-901, Brazil

## Abstract

As progressive organ shortage in cardiac transplantation demands extension of donor criteria, effort is needed to optimize graft survival. Reactive oxygen and nitrogen species, generated during organ procurement, transplantation, and reperfusion, contribute to acute and late graft dysfunction. The combined application of diverse substances acting via different molecular pathways appears to be a reasonable approach to face the complex mechanism of ischemia reperfusion injury. Thus, an antioxidant solution containing *α*-ketoglutaric acid, 5-hydroxymethylfurfural, *N*-acetyl-L-methionine, and *N*-acetyl-selenium-L-methionine was combined with endogenous angiotensin-(1-7). Its capacity of myocardial protection was investigated in isolated Langendorff-perfused rat hearts subjected to warm and cold ischemia. The physiological cardiac parameters were assessed throughout the experiments. Effects were evaluated via determination of the oxidative stress parameters malondialdehyde and carbonyl proteins as well as immunohistochemical and ultrastructural tissue analyses. It was shown that a combination of 20% (*v*/*v*) antioxidant solution and 220 pM angiotensin-(1-7) led to the best results with a preservation of heart tissue against oxidative stress and morphological alteration. Additionally, immediate cardiac recovery (after warm ischemia) and normal physiological performance (after cold ischemia) were recorded. Overall, the results of this study indicate substantial cardioprotection of the novel combination with promising prospective for future clinical use.

## 1. Introduction

Heart transplantation remains the most effective therapy for end-stage heart failure. Though significant advances have been made since the first transplant [1], it seems the field has “hit the wall” with cardiac transplant survival in the current era being equivalent to the prior cohort.

Additionally, the number of probable heart transplantation patients is increasing, while the number of donors remains constant. Hence, marginal donors like, for example, donors older than 55 years are accepted for older and/or seriously sick recipients. However, marginal donors are more vulnerable to ischemia [2–4]. In a time where we try to push the limits and transplant “high-risk” patients with “high-risk” donors due to the mentioned organ shortage, efforts must be made to optimize the success of each transplant [5, 6]. In organ transplantation, early dysfunction and late dysfunction remain major limiting factors for long-term graft survival [6]. During the transplantation process, organs undergo several antigen-independent insults, which may trigger or amplify subsequent host responses, negatively influencing organ and patient survival. These effects are related to transient episodes of warm and cold ischemia during organ procurement, followed by ischemia-reperfusion injury (IRI) [7]. One of the underlying mechanisms of myocardial IRI is the generation of reactive oxygen and nitrogen species (RONS), such as hydroxyl radical (^·^OH) and peroxynitrite (ONOO^−^), which are potent oxidizing radicals in vascular cells [8, 9]. Various animal studies on IRI have been performed, by either enhancing the endogenous defense mechanisms or by administrating substances with free radical scavenging abilities [10–12]. Few led to advances in preservation solutions [13]. The antioxidant-based strategy has recently shown improvement of posttransplant cardiac function after adult [14] and pediatric heart transplantation [15] through low-dose dopamine donor pretreatment in the clinical setting.

Therefore, the approach in this study was the investigation of a combination of an antioxidative solution and angiotensin-(1-7) (Ang 1-7) regarding its possible positive effects against IRI. Ang 1-7 is an endogenous peptide of the renin-angiotensin system, acting via the MAS receptor [16]. Beside the classical RAS, which contains the ACE-Ang II-AT1-receptor pathway and leads to vasoconstriction and retention of sodium/water, there is the alternative RAS. It consists of the ACE2-Ang 1-7-MAS-receptor axis, with effects opponent to those of the classical pathway like vasodilatation, antifibrosis, and antiproliferation [17–19]. Considering the heart, a concentration-dependent effect of Ang 1-7 was found in arrhythmias: 220 pmol Ang 1-7/L displayed an antiarrhythmic effect, while a 10-fold higher amount led to proarrhythmic impacts [19]. In studies regarding IRI, Ang 1-7 showed cardioprotective effects, for example, to reduce IRI-induced cardiac arrhythmias [20, 21], to improve postischemic heart function [22], and to have antiremodeling [23] and vasodilatative effects [24]. Its clinical potential is shown in ongoing phase I studies using a patented Ang 1-7 preparation [16]. An intravenous formulation of Ang 1-7 has also been successfully tested in preeclamptic patients with significant improvement in endothelial function [25].

The applied antioxidant solution consists of *α*-ketoglutaric acid (*α*-KG), 5-hydroxymethylfurfural (5-HMF), *N*-acetyl-selenium-L-methionine (NASeLM), and *N*-acetyl-L-methionine (NALM). *α*-KG is an endogenous intermediate of the citric cycle with pleiotropic activity in the metabolic and cellular pathways, for example, in regulation of amino acid synthesis, ATP production, and NAD^+^/NADH generation (and thereby influencing ROS levels) [26]. Basic research showed that conditions of tissue hypoxia led to an increase of *α*-KG serum levels, resulting in cardioprotection via the kynurenic acid pathway [27]. 5-HMF was recently reported as an in vitro free radical scavenger, on the one hand capable of upregulating the transcription of antioxidative acting enzymes [28], on the other hand modulating the base excision repair pathway [29]. NALM and NASeLM are added for methionine and selenium supply, as they play also an essential role in the body's antioxidant defense system [30–33]. The applied solution has recently been investigated and showed antioxidative effects during ischemia in rat hearts [34].

In this study, the antioxidative solution was combined with Ang 1-7 and evaluated considering its efficacy on cardiac function and myocardial protection. The hypothesis is that perfusing hearts with the previously mentioned cardioprotective agents, which act via different molecular pathways, will reduce acute cardiac manifestations of IRI. Two experimental models, a regional ischemia reperfusion model (warm ischemia) and a global ischemia reperfusion model (cold ischemia), were used. In the first stage of the study, the aim was to investigate effects of four different test solutions during IRI. Therefore, warm ischemia was applied in order to induce high levels of oxidative stress in a short period of time. Finally, the test solution with the most effective cardioprotection in terms of IRI arrhythmias, coronary flow, systolic and diastolic tension, and oxidative stress has been chosen and translated to an experimental setting of cold ischemia in order to simulate heart transplantation conditions.

## 2. Materials and Methods

### 2.1. Animals

Male Wistar rats, 3 months old, 250-350 g, were obtained from Medical University of Vienna (Department of Biomedical Research). For cold ischemia, 12-14-week-old male Wistar rats between 250 and 350 g were provided from the animal facility of the Biological Sciences Institute, Federal University of Minas Gerais, Brazil. All rats were maintained under standardized conditions with an artificial 12 h light/12 h dark cycle and free access to standard chow and water *ad libitum*. All studies in animals were conducted in compliance with the Austrian law on experimentation with laboratory animals, the International Guiding Principles for Biomedical Research Involving Animals, and the National Ethic Committee of the Federal University of Minas Gerais (CETEA; protocol number 148/07).

### 2.2. Reagents and Chemicals

HPLC-grade deionized water was applied during all experiments including Langendorff studies. All chemicals and reagents were of analytical grade and used without further purification.

NaCl, KCl, KH_2_PO_4_, MgSO_4_, CaCl_2_, NaHCO_3_, and dextrose were purchased from Roth (Karlsruhe, Germany). *α*-KG, 5-HMF, NALM, NASeLM, and glucose were supplied by CYL-Pharma (Lassnitzhöhe, Austria). Human albumin solution 20% was provided from Behring (Marburg, Germany). Acetonitrile, NH_4_CH_3_COO, 1-butanol, ethanol, HPLC-grade water, and HCl were obtained from Merck (Darmstadt, Germany). 2-Thiobarbituric acid, butylated hydroxytoluene, ethyl acetate, trichloroacetic acid solution, 2,4-dinitrophenylhydrazine (DNPH), malondialdehyde tetrabutylammonium salt, guanidine hydrochloride, and tris(hydroxymethyl) aminomethane were supplied by Sigma-Aldrich (Vienna, Austria).

### 2.3. Apparatus

Warm and cold ischemia experiments were carried out with the Harvard Apparatus IH-5 Langendorff and the HSE-isoheart software.

HPLC analysis of malondialdehyde (MDA) and carbonyl proteins (CPs) was performed with an Agilent Technologies 1260 Infinity Quat Pump VL and a Merck-Hitachi LaChrom L-7400 UV detector. A LiChroCART® RP-18 endcapped (5 *μ*m) column (250-4) was used with a mobile phase consisting of ammonium acetate buffer (100 mM) and acetonitrile (50 + 50 (*v*/*v*)) at a flow rate of 1 mL/min. All finally prepared samples were injected via a 100 *μ*L loop. The detection wavelength was set to 532 nm for MDA detection and to 370 nm for CP detection. Agilent ChemStation software was used for the evaluation of peak areas.

### 2.4. Preparation of Applied Solutions

Four different solutions were prepared: Krebs-Ringer solution (KRS) as the negative control, angiotensin-(1-7) solution (Ang), a solution containing 5% (*v*/*v*) multicomponent solution and Ang 1-7 (San 5), and a solution consisting of 20% (*v*/*v*) multicomponent solution and Ang 1-7 (San 20). These solutions were prepared as follows: To obtain Krebs-Ringer stock solution, 2.37 M NaCl, 0.094 M KCl, 0.024 M KH_2_PO_4_, 0.023 M MgSO_4_, and 0.05 M CaCl_2_ were mixed. For the finally applied KRS, 100 mL Krebs-Ringer stock solution, 0.05 M NaHCO_3_, and 0.023 M dextrose were mixed with water ad 2 L. The test solution Ang was obtained by dissolving 0.22 nM Ang 1-7 in KRS. The multicomponent solution was prepared with 62 mM *α*-KG, 24 mM 5-HMF, 166 mM glucose, 0.5 mM NALM, and 0.008 mM NASeLM. This composition has been chosen with regard to pharmacological adverse effects based on preliminary investigations. Finally, the solution was adjusted to a pH value of 5.7 with 0.1 M NaOH. For the application in Langendorff experiments, the multicomponent solution was diluted with KRS either 5 + 95 (*v*/*v*) for San 5 or 20 + 80 (*v*/*v*) for San 20. Both San 5 and San 20 contained additionally Ang 1-7 at a concentration of 0.22 nM. This dosage was found to be the most effective in other studies [19].

All applied solutions contained 0.02% (*v*/*v*) albumin for physiological reasons. Finally, the solutions were filtered through a 0.45 *μ*m membrane and stored at 4°C in the refrigerator.

### 2.5. Isolated Heart Preparation: Warm Ischemia through Coronary Artery Occlusion

Rats were anaesthetized with ketamine i.p. (70-100 mg/kg) and midazolam i.p. (2.5 mg/kg). The depth of analgesia and anesthesia was checked by the animals' response to a toe pinch. Thoracotomy was performed, and the heart was rapidly separated from its neighboring structures, excised, and placed in ice-cold KRS (pH 7.4) immediately before cannulation via aortic stump to the Langendorff apparatus. The perfusion fluid was maintained at 37 ± 1°C, with a pressure of 70 mmHg ± 10 mmHg and constant oxygenation (5% CO_2_/95% O_2_). During the stabilization period, all hearts were perfused with KRS. At the end of stabilization, a latex balloon, connected to a pressure transducer, was inserted into the left ventricle. Cardiac performance was measured at a left ventricular end-diastolic pressure of 10 mmHg throughout the experiment. Coronary flow was determined by collecting the perfusate over a period of 1 minute at regular intervals.

After stabilization period, the perfusion solution was changed to either San 20, San 5, Ang, or retained KRS for 15 min. Following equilibration, the left anterior descending coronary artery was ligated according to the method described by Lubbe et al. [35], beneath the left auricular appendage together with the adjacent veins. The ligature was released after 15 minutes, and reperfusion was performed for additional 30 minutes. After 90 min, the experiment was finished and the heart was divided into a part that was immediately frozen with liquid nitrogen and stored at -80°C and a part that was stored in formalin. Frozen heart parts were used for oxidative stress marker investigation; formalin parts were applied in histological and immunohistochemical analyses. In order to get basal nonoxidized values for comparison, hearts without oxidative stress (no occlusion, no ischemia) were used. All experiments were carried out each with 5 different hearts (*n* = 5).

### 2.6. Determination of Oxidative Stress Markers

For homogenization, the hearts were frozen in liquid nitrogen and pestled afterwards. The final powdery tissue was weighed in 100 mg quantities and extracted with 1 mL Tris-HCl buffer (10 mM, pH 7.4). After vortexing and centrifugation (30 min, 6500 rpm, and 4°C), the supernatant was used for MDA and CP determination. The investigation of oxidative stress markers was carried out with HPLC/UV-Vis according to Russ et al. [36]. Briefly, MDA was determined with 2-thiobarbituric acid and detected at 532 nm; CPs were examined via DNPH and investigated at 370 nm. For accurate analysis, external standards of DNPH and MDA were daily freshly prepared and investigated.

### 2.7. Statistical Analysis

For statistical significance, *p* values were calculated with IBM Statistical Package for the Social Sciences (SPSS) statistics 25 software. For each tested parameter, all values of one group were compared to another group as independent *t*-test samples. This was repeated for all possible combinations of all groups. A calculated *p* value < 0.05 was considered statistically significant.

### 2.8. Histological and Immunohistochemical Protocols for Warm Ischemia Hearts

The hearts were fixed in 10% (*v*/*v*) neutrally buffered formalin and embedded in paraffin. From the blocks, 4 *μ*m thick sections were cut and stained with hematoxylin and eosin (HE). Further on, immunohistochemical staining with antibodies against 4-hydroxynonenal (4-HNE) (ab46545, 1 : 200, Abcam, Cambridge, MA, USA) and nitrotyrosine (A21285, 1 : 200, Thermo Fisher Scientific, Waltham, MA, USA) was performed. 4-HNE was stained on DAKO Autostainer (DAKO, Glostrup, Denmark), after pretreatment with Natrium-Citrat-Puffer pH 6.0 for 40 min at 150 W. For detection, EnVision Kit 5007 (DAKO, Glostrup, Denmark) and 3,3′-diaminobenzidine (DAB) were used. Nitrotyrosine stain was performed using the ultraView detection kit (Ventana) on BenchMark ULTRA slide staining instrument (Ventana). After pretreatment with CC1 for 80 min, DAB substrate-chromogen was used for visualization. Immunostained sections were analyzed and evaluated on the basis of the average intensity of staining as negative or positive (weak, intermediate, or strong).

### 2.9. Isolated Heart Preparation: Cold Ischemia Induced by Celsior® Cardioplegia

To provide conditions most similar to the process of organ transplantation, the following procedures were carried out: Laparotomy was performed in rats previously anaesthetized with 0.1 mL/100 mg thiopental sodium (CRISTALIA, Brazil). The inferior vena cava was exposed, and 400 IU of heparin (ROCHE, Brazil) was administered intravenously, using an insulin syringe. After one minute, the inferior vena cava and abdominal aorta were cut for exsanguination. Thoracotomy was performed rapidly, the thymus removed gently, and the superior vena cava cut 1-2 mm before its confluence into the right atrium. Warm blood was removed from the thoracic cavity by using 1-2 pieces of gauze. The brachiocephalic trunk was clamped 2-3 mm after its outlet from the aortic arch using a curved hemostat. A second hemostat was attached between the outlet of the brachiocephalic trunk and the left carotid artery.

Cold cardioplegic solution (Celsior®, IMSTIX SANGSTADT, France) was applied via the brachiocephalic trunk using a subcutaneous needle, prior bent to a 90° angle, attached to a 5 mL syringe. Cardioplegia was performed air-free and under constant pressure (5 mL/min) to avoid volume overload. Simultaneously, the heart was cooled from the outside using cardioplegic solution and crushed ice. After cardiac arrest, the hearts were harvested rapidly by cutting off the abdominal aorta, the inferior vena cava 1-2 mm before its confluence with the right atrium, and both lungs. The hearts were stored in Celsior® solution at 4°C for 6 hours before being attached to the Langendorff perfusion system via an aortic stump, retrograde perfused with 37 ± 1°C warm KRS at a constant perfusion pressure of 70 mmHg and continuously oxygenated with 5%CO_2_ + 95%O_2_. After a short period of stabilization (10 minutes, KRS perfusion), the hearts were perfused for 60 minutes with KRS or San 20. All groups were performed with 10 different hearts (*n* = 10). Immediately after reperfusion, the left ventricle was harvested and fixated for histology and scanning electron microscopy.

### 2.10. Histological Protocols for Cold Ischemia Hearts

For histological examination, left ventricle tissue blocks were fixated in 2.5% glutaraldehyde and 2% paraformaldehyde in 0.1 mol/L cacodylate buffer of a pH of 7.2, embedded in historesin (LKB, Bromma, Sweden), and cut to obtain 4 *μ*m sections, which were stained with a mixture of methylene blue-azure II and basic fuchsin [37]. For ultrastructural examination, rat hearts were fixated in a mixture of 2.5% glutaraldehyde and 2% paraformaldehyde, postfixated in 2% OsO4, dehydrated, critical point dried, and gold sputtered. Representative electron micrographs were captured using a DSM 950 (Zeiss). Both groups were compared to a native, freshly excised rat heart.

## 3. Results

### 3.1. Warm Ischemia through Coronary Artery Occlusion

#### 3.1.1. Physiological Langendorff Parameters

**Figure 1:**
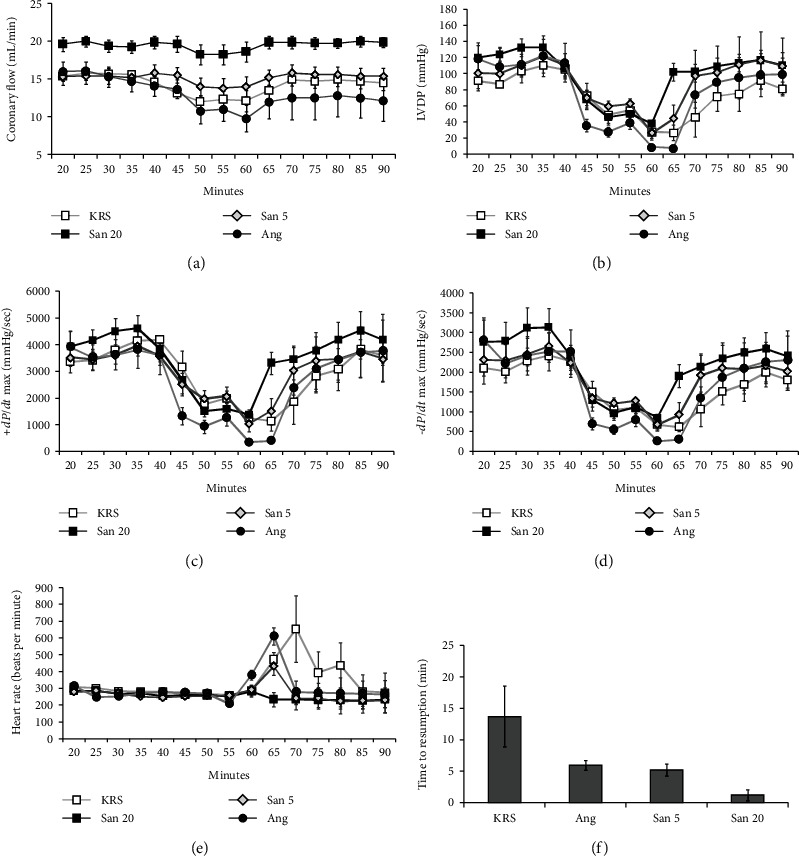
Comparison of coronary flow (a), LVDP (b), +*dP*/*dt*_max_ (c), −*dP*/*dt*_max_ (d), heart rate (e), and time to resumption (f) of rat hearts during warm ischemia experiments with four different treatments. These studies were performed with a period of 35 minutes of stabilization/perfusion, an interval of 15 minutes of regional ischemia, and following 30 minutes of reperfusion. Five different hearts were tested in each group (*n* = 5). The error bars represent the standard error of the mean in each group.

Four different groups (KRS, Ang, San 5, and San 20) were tested in warm ischemia experiments. The group receiving San 20 showed a significant increase in coronary flow, compared to the groups treated with San 5, Ang, or KRS. Additionally, the decrease of the coronary flow during occlusion (minute 45-60) was lower in all groups containing multicomponent solution (San 5 and San 20). A comparison of the coronary flow alteration of all groups during warm ischemia is shown in Figure 1(a). For functional cardiac parameters measured at Langendorff, the best results were again achieved with San 20. Systolic tension was mostly enhanced, and diastolic tension was best preserved in this group compared to the control group receiving KRS. San 20 hearts showed an almost immediate, excellent recovery after the occlusion of a coronary artery considering LVDP and ±*dP*/*dt*_max_ (Figures 1(b)–1(d)). As shown in Figure 1(e), hearts treated with San 20 showed a normal heart rate of about 300 bpm in reperfusion whereas in other groups (San 5, Ang, and KRS), postischemic arrhythmias were observed. Appropriately, the San 20 group achieved the fastest normal sinus rhythm after occlusion as can be seen in Figure 1(f).

#### 3.1.2. Oxidative Stress on Hearts with Warm Ischemia

**Figure 2:**
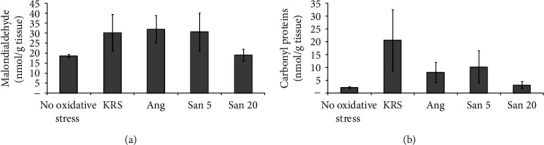
Oxidative stress in different treated rat hearts after warm ischemia experiments determined via MDA (a) and CPs (b). Five different hearts were investigated in each group (*n* = 5). The error bars represent the standard deviation in each group. Compared to a no-ischemia control, San 20 displayed no statistically significant rise (*p* value > 0.05) in oxidative stress parameters, whereas all other groups showed *p* values lower than 0.04 (significant).

For the investigation of the occurred oxidative stress through warm ischemia in the different treated hearts, MDA and CPs were determined via HPLC/UV-Vis.  Figure 2 depicts the results of these investigations in nmol/g tissue for MDA (a) and CPs (b). It can be seen that the values of the San 20 group are comparable with the values of the hearts without oxidative stress. All other groups showed an increase of oxidative stress parameters. With regard to MDA, the rise in the KRS, Ang, and San 5 groups is comparable, whereas the increase of CPs was similar in the Ang and San 5 groups and highest in the KRS group. *p* values were calculated comparing hearts without oxidative stress and the different treated heart groups. The KRS, Ang, and San 5 groups showed a statistically significant rise compared to hearts without oxidative stress in both MDA and CPs with *p* values < 0.04. The San 20 group on the other hand led to no significant rise in both oxidative stress parameters compared to no-stress values. Overall, these results indicate a significant positive, antioxidative effect of San 20.

#### 3.1.3. Histological and Immunohistochemical Analyses

**Figure 3:**
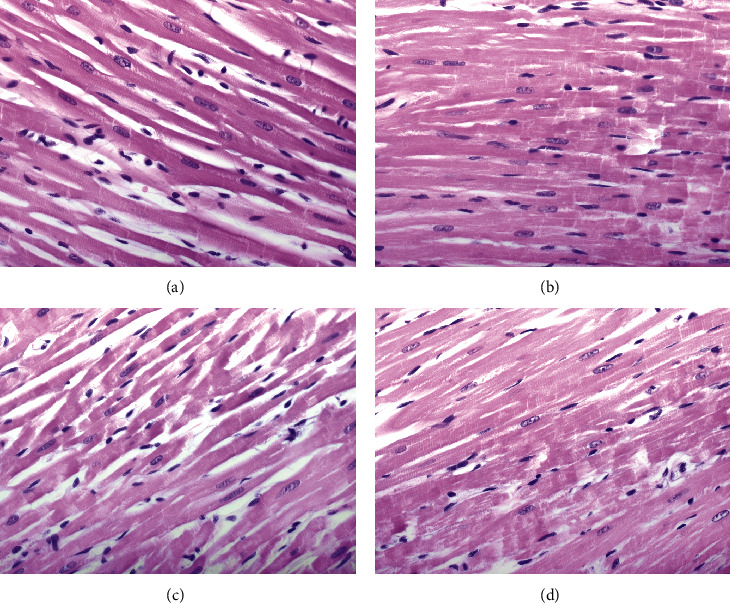
HE staining of different treated rat hearts after warm ischemia experiments: San 20 (a), San 5 (b), Ang (c), and KRS (d). Histological findings showed myocytes with enlarged, oval, centrally located nuclei in all groups. The used original magnification is 400x.

Histologically, rat heart tissue treated with San 20, San 5, Ang, or KRS and stained with HE showed in all groups myocytes with enlarged centralized nuclei (Figure 3).

**Figure 4:**
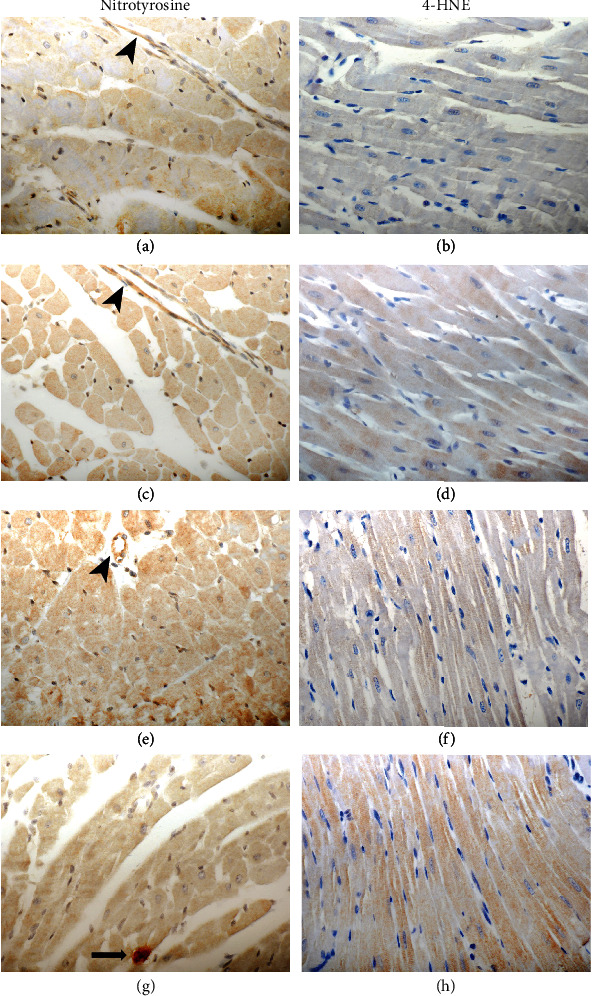
Comparison of immunohistochemical findings after warm ischemia experiments. Nitrotyrosine and 4-HNE staining in the myocytes is less expressed in the San 20 (a, b) group when compared to San 5 (c, d), Ang (e, f), and KRS (g, h). Note positive endothelial cells (arrowhead: images (a), (c), and (e)), and a mastocyte (arrow: image (g)) as positive internal control. Original magnification 400x.

Additional immunohistochemical analysis with antibodies against nitrotyrosine and 4-HNE showed weak intensity of staining in the San 20 group, while the staining in other groups was weak to intermediate (Figure 4).

### 3.2. Cold Ischemia Induced by Celsior® Cardioplegia

**Figure 5:**
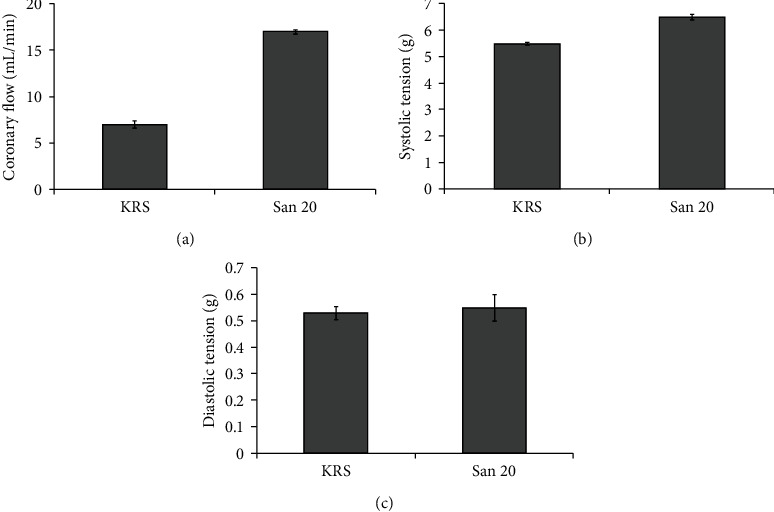
Comparison of average coronary flow (a), systolic tension (b), and diastolic tension (c) in two different treated groups of rat hearts after cold ischemia. In each group, ten different hearts were used (*n* = 10). The error bars represent the standard deviation in each group. Compared to each other, there was no statistically significant difference in diastolic tension but a distinct significant higher coronary flow and systolic tension in the San 20 group.

Again, the group receiving San 20 (the combination of 20% (*v*/*v*) antioxidative solution and Ang 1-7) presented a significant increase of the coronary flow compared to the KRS control group (Figure 5(a)). Also concerning systolic tension (Figure 5(b)), the San 20 group was significantly higher, whereas the diastolic tension however was not significantly different within both groups (Figure 5(c)).

#### 3.2.1. Histological Analysis

**Figure 6:**
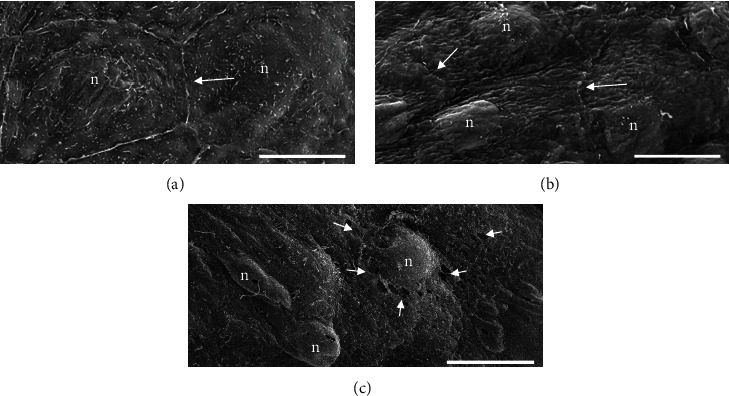
Scanning electron micrographs on the endothelial layer lining the endocard of rat hearts after cold ischemia. The endothelial cells of rat hearts immediately sacrificed without cardioplegia and perfusion (a) show a polygonal morphology and exhibit numerous microvilli at their cell surface. The contact zones between the cells (arrows) are clearly visible. The endothelial cells of rat hearts perfused with San 20 (b) have a similar polygonal-shaped appearance with a varying number of microvilli. Cells treated with Krebs-Ringer solution (c) are partially damaged as documented by leaks within the cell membrane (arrowheads). The central zone contains a prominent nucleus (n). The peripheral margins are attenuated. Bar = 10 *μ*m.

At the ultrastructural level, the morphology of endothelial cells of freshly excised and immediately fixed hearts (Figure 6(a)) closely resembled to endothelial cells of hearts perfused with San 20 (Figure 6(b)). The endothelial cells of both groups showed a polygonal-shaped morphology with numerous microvilli at the cell surface and clearly visible contact zones between the cells. The endothelial cells of hearts perfused with KRS exhibited a smoother cell surface with only few microvilli (Figure 6(c)).

**Figure 7:**
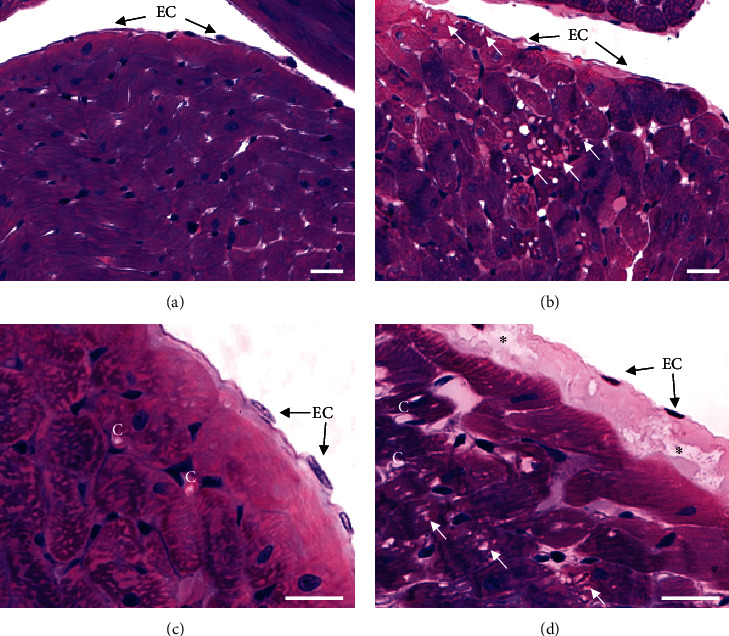
Light microscopy photographs on endo- and myocard of rat hearts after cold ischemia stained with a mixture of methylene blue-azure II and basic fuchsin. Hearts perfused with San 20 (a, c) show morphological details characteristic of a normal endo- and myocard. In contrast, hearts perfused with Krebs-Ringer solution (b, d) exhibit cell damages characterized by enhanced vacuoles (white arrows) within heart muscle cells of the myocard. In addition, signs of degeneration of connective tissue underlying endothelial cells (EC) within the endocard are reflected by gaps (asterisks). Capillaries (C) are located in the connective tissue surrounding the cardiomyocytes. Bar = 25 *μ*m.

Histological examination of the left ventricle exposed to San 20 reperfusion showed intact endothelium and underlying connective tissue of the endocardium. Additionally, the heart muscle cells had a normal appearance (Figures 7(a) and 7(c)). The cardiac wall exposed to KRS (Figures 7(b) and 7(d)) contained beside areas with normal morphology, also regions with structural alterations, especially in areas of the endocardium and subendocardial heart muscle cells. In addition, the connective tissue underlying the endothelial sheet showed degenerating processes reflected by gaps. Some cardiomyocytes showed varying degrees of disintegration up to myocytolysis reflected by vacuolization.

## 4. Discussion

For cardiac transplantation, optimizing of graft survival is a high priority. IRI is an important contributor to early graft failure and is caused by ROS and RNS, generated during organ procurement, transplantation, and reperfusion. Hence, this is a much-studied area and of high importance to the heart transplantation field [2–4].

From the numerous experimental models, only few observations manage to be translated into the clinical setting. Though there are inherent shortcomings of ex vivo basic animal studies, they are indispensable in determining appropriate drug dosages and effectiveness under controlled laboratory settings. With their strength of reproducibility and cost-effectiveness, they constitute the first step in developing therapies for future clinical use.

In this study, the myocardial protection of an antioxidative solution combined with Ang 1-7 during IRI has been investigated by Langendorff via applying two experimental models, a regional ischemia-reperfusion model and a global ischemia-reperfusion model. The regional ischemia model (warm ischemia through coronary artery occlusion), a well-established model, was investigated first due to massive oxidative stress generation in a short experimental time of 90 minutes. For simulating transplantation conditions, the solution presenting the strongest positive effect during warm ischemia, which was San 20, was chosen for the protocol of cold ischemia. Global (cold) ischemia was achieved through cardioplegia with Celsior® solution. Reperfusion was examined with San 20, and the postconditioning effect was compared with KRS (negative control). Beside the measured Langendorff parameters, heart tissues were investigated considering occurred oxidative stress via oxidative stress parameters and histological and immunohistological analyses.

Applying San 20 during warm ischemia led to an improvement compared to the negative control (KRS). Measured Langendorff parameters (CF, LVDP, ±*dP*/*dt*_max_, and time to resumption) were least affected through coronary artery ligature when perfusing the hearts with San 20. Regarding time to resumption (TTR), it was also observed that Ang 1-7 alone is able to examine positive effects during IRI. KRS-treated rat hearts (negative control) needed 14 minutes on average to get back to a normal sinus rhythm, while in the Ang group, TTR was reduced to about 6 minutes. A similar TTR was found in the San 5 group. This may indicate that this positive effect in reperfusion is assigned to Ang 1-7, which thereby contributes to cardioprotection. It seems that the most likely mechanism of the observed cardioprotective effect of Ang 1-7 in this study can be attributed to its antiarrhythmic effect. Numerous experimental and clinical studies have demonstrated the beneficial effects of the ACE2/angiotensin-(1-7) axis on heart failure development and elucidated its pharmacological and therapeutic potential. The binding of Ang 1-7 to its selective MAS receptor counteracts the deleterious effects evoked by angiotensin II [38, 39]. The antiarrhythmic effect of Ang 1-7 itself has been described in different studies [20, 21, 40, 41]. However, a combination with 20% antioxidative solution (San 20) led to better results with the lowest observed TTR of about 1 minute only.

Considering oxidative stress, the generally accepted parameters MDA and CPs were chosen as they provide essential information on cell decay of cardiomyocytes via oxidative lipid and protein damage. When warm ischemia in rat hearts was treated with San 20, these markers stayed at the same level as in hearts without oxidative stress, indicating a positive radical scavenging effect and in further consequence minimizing tissue damage. When comparing nonoxidative stress hearts with all other treated hearts exposed to oxidative stress, MDA amount was nearly doubled in the KRS, San 5, and Ang groups and CP amount rose up to 10-fold in the KRS group (negative control) and was doubled in the San 5 and Ang groups. Although the detected CP amount was lower in the San 5 and Ang groups compared to the KRS group, this effect was not statistically significant. Overall, the treatment with San 20 resulted in nonappearance of investigated oxidative stress markers despite occlusion of a coronary artery. The results of these experiments indicate that Ang 1-7 showed no distinct antioxidative effect in these studies and that the positive effect of the combined solution is dose dependent. Additionally, the supplemental immunostaining findings supported these results. Nitrotyrosine and 4-HNE staining was less expressed in the San 20 group when compared to all other groups.

The same trend of improvement was found in cold ischemia experiments. San 20-treated hearts showed a higher coronary flow, as well as systolic tension despite 6 hours of cold ischemia compared to a negative control. In histological analysis of these hearts, San 20 hearts resembled the structure of freshly hearts without oxidative stress whereas the negative control showed structural alteration.

Although these investigations are basic research with short time scale limits of the applied models, they cover different points like pretreatment, ischemia/reperfusion, postconditioning, and overall performance. In order to realize the potential of this new approach and before applying this novel combination to humans, further investigations, for example, in pig hearts have to be performed. Overall, the results of this study indicated a distinct myocardial protection of San 20 during IRI in rat hearts. It seems that both components of the combination—Ang 1-7 and antioxidative solution—support each other in their positive effects. Thus, the combination of both—acting via antiarrhythmic and antioxidative mechanisms—is a useful approach to oppose negative effects of IRI.

## 5. Conclusions

In recent years, much attention has been given to the underlying mechanisms of IRI, showing the complexity of its various molecular pathways. Therefore, such a scenario demands targeting different molecular mechanisms, which were approached in this study by a combination of Ang 1-7 with an antioxidative solution consisting of four pharmacologically active compounds. According to the outcome of this work, this novel combination seems to be a promising attempt for future clinical use.

## Data Availability

All the data used to support the findings of this study are available from the corresponding author upon request.
